# Immune Microenvironment Related Competitive Endogenous RNA Network as Powerful Predictors for Melanoma Prognosis Based on WGCNA Analysis

**DOI:** 10.3389/fonc.2020.577072

**Published:** 2020-10-27

**Authors:** Yaqi Cheng, Chengxiu Liu, Yurun Liu, Yaru Su, Shoubi Wang, Lin Jin, Qi Wan, Ying Liu, Chaoyang Li, Xuan Sang, Liu Yang, Chang Liu, Xiaoran Wang, Zhichong Wang

**Affiliations:** ^1^State Key Laboratory of Ophthalmology, Zhongshan Ophthalmic Center, Sun Yat-sen University, Guangzhou, China; ^2^Department of Ophthalmology, Afﬁliated Hospital of Qingdao University Medical College, Qingdao, China

**Keywords:** skin melanoma, weighted gene co-expression network analysis, ceRNA network, tumor immune microenvironment, prognosis

## Abstract

Cutaneous melanoma is the most life-threatening skin malignant tumor due to its increasing metastasis and mortality rate. The abnormal competitive endogenous RNA network promotes the development of tumors and becomes biomarkers for the prognosis of various tumors. At the same time, the tumor immune microenvironment (TIME) is of great significance for tumor outcome and prognosis. From the perspective of TIME and ceRNA network, this study aims to explain the prognostic factors of cutaneous melanoma systematically and find novel and powerful biomarkers for target therapies. We obtained the transcriptome data of cutaneous melanoma from The Cancer Genome Atlas (TCGA) database, 3 survival-related mRNAs co-expression modules and 2 survival-related lncRNAs co-expression modules were identified through weighted gene co-expression network analysis (WCGNA), and 144 prognostic miRNAs were screened out by univariate Cox proportional hazard regression. Cox regression model and Kaplan-Meier survival analysis were employed to identify 4 hub prognostic mRNAs, and the prognostic ceRNA network consisting of 7 lncRNAs, 1 miRNA and 4 mRNAs was established. After analyzing the composition and proportion of total immune cells in cutaneous melanoma microenvironment through CIBERSORT algorithm, it is found through correlation analysis that lncRNA-TUG1 in the ceRNA network was closely related to the TIME. In this study, we first established cutaneous melanoma’s TIME-related ceRNA network by WGCNA. Cutaneous melanoma prognostic markers have been identified from multiple levels, which has important guiding significance for clinical diagnosis, treatment, and further scientific research on cutaneous melanoma.

## Introduction

Cutaneous melanoma is the most malignant skin tumor originating from melanocytes with abnormal proliferation and differentiation, and is the leading cause of skin cancer-related deaths (1). The incidence of melanoma is increasing year by year, and it is characterized by high recurrence rate, high mortality rate, and high drug resistance (2, 3). Early melanoma has a higher cure rate after complete resection, and the mortality rate of stage III/IV patients is as high as 70%, and the 5-year survival rate is less than 16% (4). In recent years, targeted therapy and immunotherapy for melanoma have made certain breakthroughs, while low response rates and severe adverse reactions have limited its long-term effects (5, 6). Therefore, seeking in-depth understanding of the pathogenesis and malignant transformation mechanism of melanoma, exploring more biological targets, and making accurate prediction of the prognosis, will bring new hope for overcoming melanoma’s recurrence and resistance, and improving the survival rate of patients.

Tumor immune microenvironment (TIME) plays an important role in tumorigenesis and therapeutic response. Melanoma cells interact with innate and acquired immune systems, resulting in the inactivation of immune cells with tumor monitoring and killing functions, the activation of tumor protective immune cells, and the formation of TIME with abnormal components and functions, which mediates the immune escape and early metastasis of melanoma (7–9). In order to break the abnormal TIME and mobilize autoimmunity to fight against melanoma, immunotherapy represented by PD-1 and CTLA-4 monoclonal antibody have been worked out, some of which partially delayed the progression of tumor. However, there are still more than half of the patients showing rapid resistance within 6-12 months and even dying of treatment-related adverse drug reactions (10). To clarify the complex tumor-immune regulation in the progression and drug resistance of melanoma, and to find new immunetherapy targets is the only way to to discover new treatment for advanced-stage, recurrent and metastatic melanoma.

Competitive endogenous RNA (ceRNA) network is the emerging post-transcriptional regulation theory in recent years: the non-coding microRNAs (miRNAs) in cells can bind to mRNAs with coding function, resulting in inactivation for translation, unstable structure and rapid degradation of binded mRNAs. At the same time, a variety of other non-coding RNAs (ncRNAs), such as circular RNA (circRNA), long non-coding RNA (lncRNA), and pseudogenes,etc. can sponge absorb miRNA, isolating it from mRNA and forming a competing relationship between ncRNAs and mRNAs, and jointly participating in the endogenous RNA regulatory network, affecting gene translation process (11). CeRNA network regulates the development of most tumors including melanoma (12). Many studies have revealed the specific mechanism by which lncRNA sponge adsorbed miRNA to regulate mRNA expression in melanoma, and clarified the role of various lncRNA-related ceRNA networks in melanoma cells’ proliferation, apoptosis, invasion and migration (13, 14). At the same time, benefiting from the development of high-throughput sequencing technology, more and more studies attempt to outline the post-transcriptional regulation map through systematically analyzing melanoma -related ceRNA networks, with the purpose of seeking for tumor-related prognostic markers (15).

Weighted gene co-expression network analysis (WGCNA) is a method of clustering genes according to expression patterns, systematically analyzing the relationship between gene modules and traits, and classifying gene functions (16, 17). WGCNA method has been widely used in the bioinformatics research on prognostic markers and therapeutic targets of tumors and other diseases.

In order to explore the role of TIME related ceRNA network in melanoma, WGCNA method was used in this study to screen the hub mRNA and lncRNA modules that were significantly related to clinical prognosis from the melanoma transcriptome data of The Cancer Genome Atlas (TCGA) database. The cox survival predicting model was established to screen the hub RNAs and construct the prognostic lncRNA-miRNA-mRNA ceRNA network. mRNAs profiles from Gene Expression Omnibus (GEO) database was used as validation set. We further analyzed the infiltrating immune cells in melanoma samples through The Cell Type Identification by Estimating Relative Subsets of RNA Transcripts (CIBERSORT) and obtained the phenotype and proportion of immune cells in TIME. Finally, the correlation between immune cell components and prognostic ceRNA network was analyzed. Our research results pointed out the possibility of TIME-associated ceRNA serving as prognostic markers for melanoma, and elucidated the mechanism of melanoma occurrence and development in combination with tumor microenvironment and post-transcriptional regulation.

## Materials and Methods

### RNA Expression Profiles and Clinical Data of Melanoma Patients

We obtained 471 cutaneous melanoma samples’ mRNA and lncRNA expression profiles, and 450 cutaneous melanoma samples’ miRNA expression profiles from the TCGA database (https://portal.gdc.cancer.gov/). 51 melanoma samples’ mRNA expression profileswere obtained from the mRNA microarray GSE98394 (GPL16791 Illumina HiSeq 2500) in GEO database. Meanwhile, the clinical information of melanoma patients was obtained from TCGA. After the collation of clinical data, 352 cutaneous melanoma samples from 350 patients with complete information of survival time, survival status, and TNM stage were selected for mRNAs and lncRNAs WGCNA analysis. MiRNA expression profiles of 440 melanoma samples with both survival time and survival status information were selected for univariate COX analysis.

### Construction of WGCNA Co-Expression Modules and Identifying Prognostic RNAs

WGCNA is an analytical method to identify gene co-expression networks based on topological overlap. The clusters (modules) of highly interconnected genes were determined by hierarchical clustering based on the connectivity and covariation coefficients of the genes. Eigengene expression patterns within each module are condensed into a “Module eigengene (ME).” MEs in the same cluster are considered to have high correlation, consistent expression patterns, and similar biological functions, which is helpful to further explore the functions of different clusters (18). We used the “goodSamplesGenes” function in the “WGCNA” R package to check the missing values of gene expression. After excluding mRNAs and lncRNAs with an average expression amount less than 1, genes with top 25% variance were performed WGCNA analysis to construct co-expression modules (19). The power value is an important soft threshold parameter for defining highly positive correlations among genes in the same module. The “WGCNA” R package was employed to test the independence and average connectivity of different modules under different power values, and the power value corresponding to an independence index of R^2^ = 0.8 was selected. The minimum numbers of genes in the mRNA module and lncRNA module were set to 50 and 30 (20). The “WGCNA” R package was used to cluster thousands of mRNAs and lncRNAs into different modules (identified by arbitrary colors). Based on clinical information, the correlation between modules and clinical phenotypes was calculated, and modules related to prognosis characters such as survival status and survival time were screened to obtain survival-related mRNAs and lncRNAs (WGCNA-mRNAs, WGCNA-lncRNAs) with P < 0.05 as the statistical significance threshold.

### Preliminary Screening for Prognostic miRNAs

Cox proportional hazard regression models were constructed with survival outcomes and survival time as dependent variables to analyze the impact of various factors on survival (21). With P < 0.05 as the statistical significance threshold, univariate Cox proportional hazard regression was employed to screen for hub miRNAs (unicox-miRNAs) related to overall survival (OS).

### mRNAs Function Annotation

Gene ontology (GO) analysis was performed to WGCNA-mRNAs to annotate the biological processes (BP), cellular component (CC), and molecular function (MF) they involved. Meanwhile, Kyoto Encyclopedia of Genes and Genomes (KEGG) analysis was used to annotate the signaling pathways associated with these WGCNA-mRNAs. These analyses were performed by the R software package “clusterProfiler”, with P < 0.05 as the threshold of statistical significance. The “ggplot2” R package was used to visualize the GO and KEGG enrichment results (22).

### lncRNA-miRNA and miRNA-mRNA Targeting Relationship Prediction

The miRcode database was used to predict the miRNAs (lnc-pre-miRNAs) targeted by WGCNA-lncRNAs (23). The Venn diagram was employed to take intersection of lnc-pre-miRNAs and unicox-miRNAs, and the mRNAs (mi-pre-mRNAs) targeted by common miRNAs were predicted by Targetscan (24) and miRDB (25) database. Intersection of mi-pre-mRNAs and WGCNA-mRNAs was taken.

### Hub Prognostic mRNAs Identifying Through Survival Prediction Model

The univariate Cox proportional hazard regression was employed to further verify the common mRNAs between WGCNA-mRNAs and mi-pre-mRNAs, and screen for hub mRNAs whose expression was related to the OS of patients. MRNAs with P < 0.05 were further evaluated by multivariate Cox proportional hazard regression to identify its ability to predict the prognosis of melanoma. The median risk score obtained through multivariate Cox regression was used as the boundary to divide melanoma patients into high- and low-risk groups. Survival analysis of high- and low-risk groups was performed through the “survival” R package and the survival curve was drawn (26). We also obtained all immunohistochemical pictures of the expression of hub genes in normal skin and melanoma samples from the Human Protein Atlas database (HPA) (http://www.proteinatlas.org), using Image J software to measure the gray-scale of all images to quantify the expression levels of these four protein, and analyzed the obtained gray-scale by GraphPad Prism version 8.0.0 for Windows (San Diego, CA, USA, http://www.graphpad.com). Difference analysis between the two groups was performed by Student’s t test and P < 0.05 was considered statistically significant (27, 28).

### Construction of Prognostic ceRNA Network

A lncRNA–miRNA–mRNA ceRNA network with hub prognostic mRNAs as the core was constructed based on the targeting relationships. This ceRNA network elucidates the competitive binding of miRNAs by lncRNAs and mRNAs, thereby revealing the complex post-transcriptional regulatory mechanisms in melanoma. Cytoscape v3.7.2 was employed to visualize the ceRNA network (29).

### Analysis of TIME Through CIBERSORT

In order to further study the specific mechanism of abnormal ceRNA network to promote tumors and explore the relationship between ceRNA and TIME, CIBERSORT algorithm was used to identify the proportion of 22 immune cells infiltrated in melanoma samples, and the analysis was repeated 100 times to improve the reliability of the results. The immune cell data of the samples with P < 0.05 were selected for the next correlation analysis (30). Pearson correlation analysis was used to verify the correlation between hub RNAs in ceRNA network and immune cell infiltration.

### Statistical Analysis

All statistical analyses were performed by R software (v.3.6.1) and the aforementioned packages.

## Results

### mRNAs in 3 Co-Expression Modules Preliminary Screened as Melanoma Survival-Related mRNAs by WGCNA Construction

**Figure 1:**
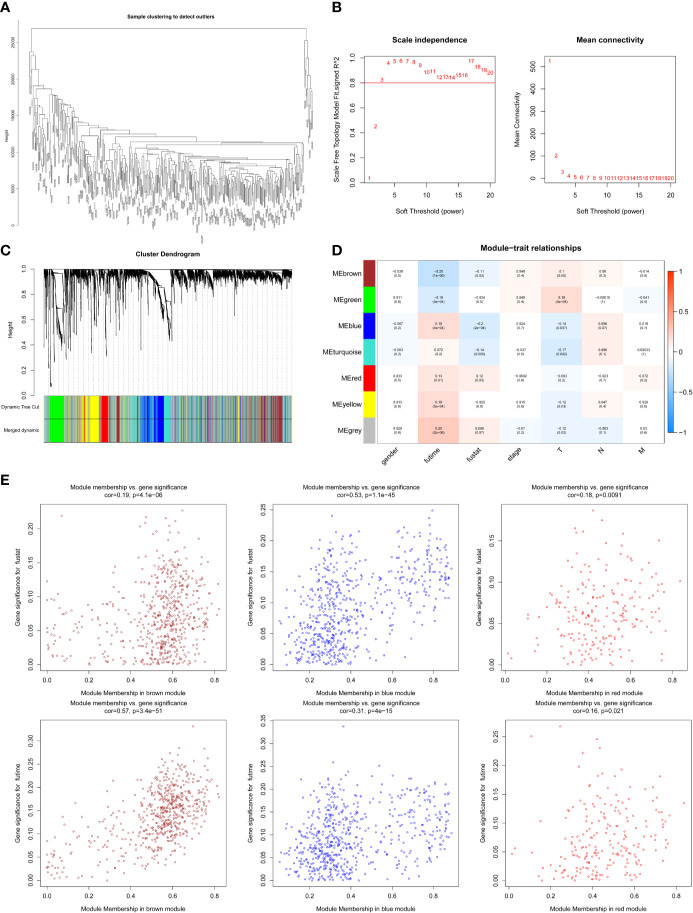
Screening survival related mRNAs by WGCNA. **(A)** Hierarchical clustering tree of 352 melanoma sample mRNAs expression patterns. **(B)** Power value screening by WGCNA. When the power value is 4, R^2^>0.8, the average connection degree <100. **(C)** Clustering and merging of mRNAs co-expression modules. **(D)** Correlation heatmap of module genes and clinical traits. Red represents positive correlation and blue represents negative correlation. The correlation increases as the the color darkens; **(E)** Scatterplot of correlation between modules and clinical traits. Brown, blue, and red mRNAs modules are positively correlated with survival status and survival time.

The expression profiles of mRNAs in 470 melanoma samples were explored, the clinic information and the therapies data of the patients were shown in **Table 1**. After preliminary sorting, we constructed the WGCNA co-expression module for 4938 mRNAs from 352 samples. Firstly, mRNAs were hierarchically clustered (**Figure 1A**), and Power values determining module independence and average connectivity were screened. When the power value is 4, the independence degree >0.8, which can better reflect the scale-free topology of the co-expression network (**Figure 1B**). Then 7 mRNAs co-expression modules were established (**Figure 1C**). The number of eigengenes contained in modules ranged from a minimum of 209 (red modules) to a maximum of 1642 (gray modules).

**Table 1:** Clinic information and the therapies data of 470 melanoma patients.

	Alive(n = 259)	Dead(n = 211)	Total(n = 470)
**Gender**			
Famale	111(42.9%)	69(32.7%)	180(38.3%)
Male	148(57.1%)	142(67.3%)	83 (61.7%)
**Breslow depth (mm)**			
t ≤ 1	34(13.1%)	25(11.9%)	59(12.6%)
1< < t ≤ 4	77(29.7%)	80(37.9%)	157(33.4%)
4 < t	83(32.1%)	60(28.4%)	143(30.4%)
Missing data	65(25.1%)	46(21.8%)	111(23.6%)
**Ulceration**			
Yes	96(37.1%)	71(33.6%)	167(35.5%)
No	75(29.0%)	71(33.6%)	146(31.1%)
Missing data	88(33.9%)	69(32.8%)	157(33.4%)
**TNM status**			
**T**			
Tis	3(1.2%)	5(2.4%)	8(1.7%)
T0	15(5.8%)	8(3.8%)	23(4.9%)
T1	30(11.6%)	12(5.7%)	42(8.9%)
T2	38(14.7%)	40(19.0%)	78(16.6%)
T3	40(15.4%)	50(23.7%)	90(19.1%)
T4	92(35.5%)	61(28.9%)	153(32.6%)
Missing data	41(15.8%)	35(16.5%)	76(16.2%)
**N**			
N0	128(49.4%)	107(50.7%)	235(50.0%)
N1	40(15.4%)	34(16.1%)	74(15.7%)
N2	28(10.8%)	21(10.0%)	49(10.4%)
N3	29(11.2%)	26(12.3%)	55(11.7%)
Missing data	34(13.0%)	23(10.9%)	57(12.1%)
**M**			
M0	227(87.6%)	191(90.5%)	418(88.9%)
M1	15(5.8%)	8(3.8%)	23(4.9%)
Missing data	17(6.6%)	10(4.7%)	2(5.7%)
**Stage**			
0	2(0.8%)	5(2.4%)	7(1.5%)
I	47(18.1%)	43(20.3%)	90(19.1%)
II	84(25.9%)	56(26.5%)	140(29.8%)
III	92(32.4%)	79(37.4%)	171(36.4%)
IV	14(5.4%)	9(4.4%)	23(4.9%)
Missing data	20(7.4%)	19(9.0%)	39(8.3%)
**Radiation therapy**			
Yes	30(11.6%)	8(3.8%)	38(8.0%)
No	196(75.7%)	51(24.2%)	247(52.6%)
Missing data	33(12.7%)	152(72%)	185(39.4%)
**Previous treatment**			
**Neoadjuvant treatment**			
Yes	11(4.2%)	14(6.6%)	25(5.3%)
No	248(95.8%)	197(93.4%)	445(94.7%)
**Interferon**			
Yes	12(4.6%)	5(2.4%)	17(3.6%)
No	7(2.7%)	17(8.0%)	24(5.1%)
Missing data	240(92.7%)	189(89.6%)	429(91.3%)
**Prior radiation therapy**		
Yes	0(0%)	0(0%)	0(0%)
No	255(98.5%)	210(99.5%)	465(98.9%)
Missing data	4(1.5%)	1(0.5%)	5(1.1%)
**Prior systemic therapy**			
Yes	15(5.8%)	17(8.1%)	32(6.8%)
No	244(94.2%)	194(91.9%)	438(93.2%)

According to the clinical information from TCGA database, the correlation between the module and clinical characters was analyzed (**Figure 1D**). It was found that brown module, blue module, and red module were highly correlated with survival time and survival status (P < 0.05, **Figure 1E**). Meanwhile, the blue module was also highly related to the T stage of melanoma (**Supplementary Figure 1A**). Therefore, it is preliminarily determined that 1401 mRNAs (WGCNA-mRNAs) in the brown, blue, and red modules are related to melanoma prognosis.

**Figure 2:**
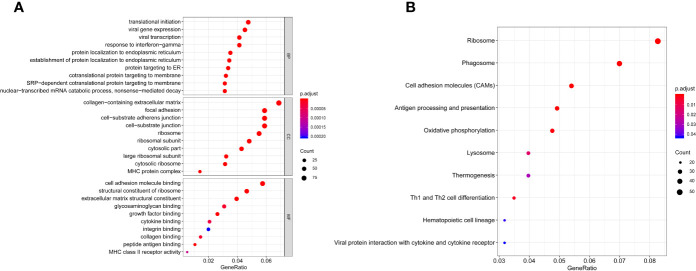
GO **(A)** and KEGG **(B)** analyses of WGCNA -mRNAs. Greater enrichment of specific functions is indicated by larger bubbles and longer columns. Red indicates smaller P values and blue indicates larger P values.

GO and KEGG analysis of 1401 WGCNA-mRNAs showed that they were mainly enriched in biological processes such as translation initiation (GO:0006413), endoplasmic reticulum localization (GO:0072599, GO:0070972), interferon gamma response (GO:0034341), extracellular matrix composition (GO:0043062, GO:0030198) (**Figure 2A** and **Table 2**), and participate in signal pathways such as ribosomes (hsa03010), antigen presentation (hsa04612), phagosomes (hsa04145), cell adhesion molecules (hsa04514), etc., which were closely related to gene translation, immune response and cell positioning (**Figure 2B** and **Table 3**). The P values of the above enrichment analysis results were all <0.05.

**Table 2:** Results of GO enrichment analysis of WGCNA-mRNAs.

Ontology	Term	Description	Count	Adj. P value
BP	GO:0006413	Translational initiation	62	5.24E-22
BP	GO:0019080	Viral gene expression	59	3.91E-20
BP	GO:0072599	Establishment of protein localization to endoplasmic reticulum	45	1.53E-18
BP	GO:0045047	Protein targeting to ER	44	1.97E-18
BP	GO:0019083	Viral transcription	54	2.34E-18
CC	GO:0044391	Ribosomal subunit	64	6.21E-26
CC	GO:0005840	Ribosome	73	4.05E-23
CC	GO:0062023	Collagen-containing extracellular matrix	91	4.45E-23
CC	GO:0022626	Cytosolic ribosome	42	3.82E-19
CC	GO:0015934	Large ribosomal subunit	43	1.18E-18
MF	GO:0003735	Structural constituent of ribosome	60	4.48E-19
MF	GO:0005201	Extracellular matrix structural constituent	51	2.31E-17
MF	GO:0019838	Growth factor binding	34	3.47E-08
MF	GO:0050839	Cell adhesion molecule binding	74	4.46E-07
MF	GO:0042605	Peptide antigen binding	14	1.47E-06

**Table 3:** Results of KEGG enrichment analysis of WGCNA-mRNAs.

TermID	Description	Gene Count	Adj. P value
hsa03010	Ribosome	52	1.22E-13
hsa04612	Antigen processing and presentation	31	4.84E-10
hsa04145	Phagosome	44	3.76E-09
hsa04514	Cell adhesion molecules (CAMs)	34	0.000153037
hsa00190	Oxidative phosphorylation	30	0.000775746
hsa04658	Th1 and Th2 cell differentiation	22	0.003212236
hsa04142	Lysosome	25	0.014674615
hsa04714	Thermogenesis	25	0.029797629
hsa04640	Hematopoietic cell lineage	20	0.042379646
hsa04061	Viral protein interaction with cytokine and cytokine receptor	20	0.043224531

### 249 lncRNAs in Brown Module Were Preliminary Screened Out as Melanoma Survival-Related by WGCNA Construction

**Figure 3:**
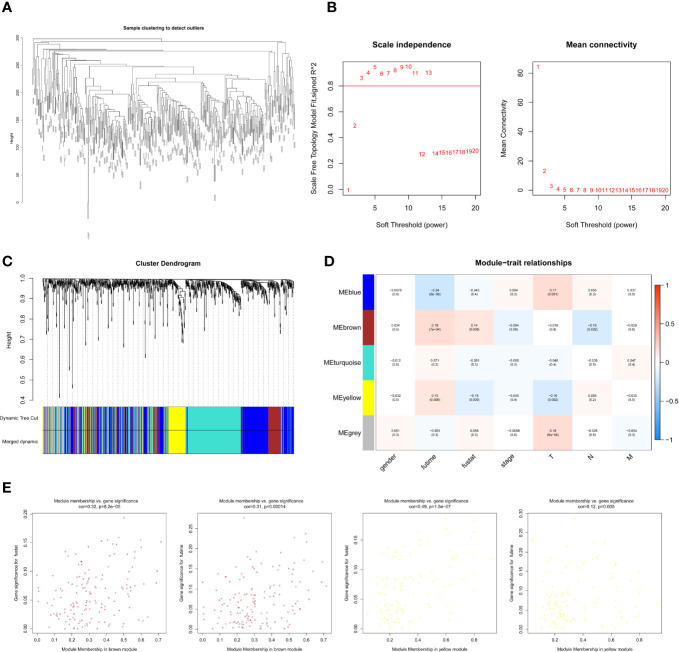
Screening survival related lncRNAs by WGCNA. **(A)** Hierarchical clustering tree of 352 melanoma sample lncRNAs expression patterns. **(B)** Power value screening by WGCNA. When the power value is 3, R^2^>0.8, the average connection degree is <100. **(C)** Clustering and merging of lncRNAs co-expression modules. **(D)** Correlation heatmap of module genes and clinical traits. Red represents positive correlation and blue represents negative correlation. The correlation increases as the the color darkens. **(E)** Scatterplot of correlation between modules and clinical traits. Brown and yellow lncRNAs modules are positively correlated with survival status and survival time.

The WCGNA co-expression modules of lncRNAs were constructed in the same way as those of mRNAs. After pre-processing the lncRNAs and clustering (**Figure 3A**), the power value was screened and determined to be 3 (**Figure 3B**). Blue (288 lncRNAs), brown (146 lncRNAs), gray (71 lncRNAs), turquoise (315 lncRNAs), and yellow (103 lncRNAs) modules were established (**Figure 3C**). The module–trait correlation analysis revealed that the brown and yellow modules were closely associated with survival time and survival status (P < 0.05, **Figures 3D, E**). The brown module was also related to the N stage of the melanoma, and the yellow module was related to the T stage (**Supplementary Figures 1B, C**). We preliminarily determined that 249 lncRNAs (WGCNA-lncRNAs) in these modules were related to melanoma prognosis.

### 144 prognostic miRNAs Were Preliminary Screened Through Univariate Cox Regression

We selected the miRNAs expression profiles of 440 samples with both survival time and survival states information for univariate proportional hazard regression. After averaging the expression levels of duplicate miRNAs and screening miRNAs with an average expression level greater than 1, we got a total of 936 miRNAs entering Cox regression. According to the statistical criteria of P < 0.05, 144 miRNAs (unicox-miRNAs), which were related to the prognosis of melanoma were screened out (**Supplementary Table 1**).

### Prediction of Competitive Relationships and Preliminary Screening 48 Hub mRNAs

**Figure 4:**
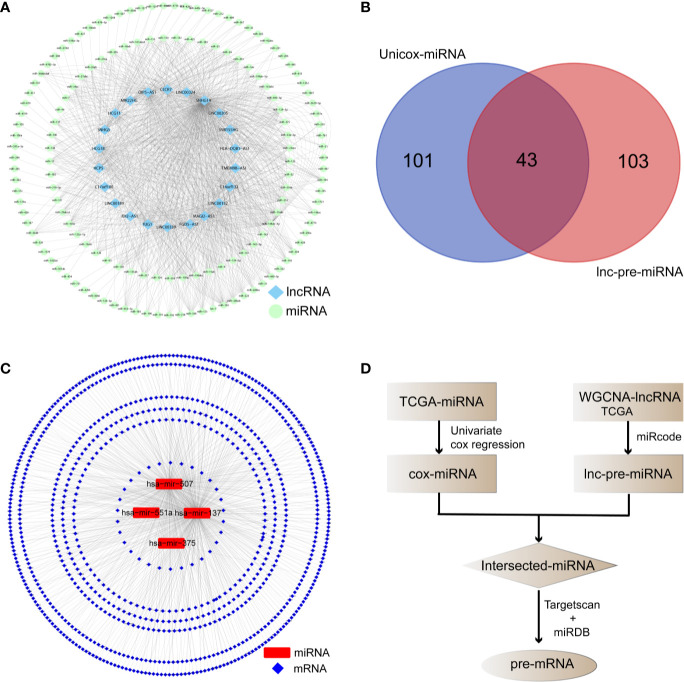
Prediction of the target relationship of lncRNAs, miRNA, and mRNA. **(A)** Target network between WGCNA-lncRNAs and lnc-pre-miRNAs. **(B)** Venn diagram was employed to take intersection of unicox-miRNAs and lnc-pre-miRNAs, through which 43 common miRNAs were obtained. **(C)** Target relationship between common miRNAs and mi-pre-mRNAs. **(D)** Flow chart of target gene prediction.

The miRcode database identified 147 lnc-pre-miRNAs targeted by the 249 WGCNA-lncRNAs, the targeting relationship is visualized in **Figure 4A**. To screen for miRNAs both involved in the broader ceRNA network and associated with melanoma prognosis, we intersected the 147 lnc-pre-miRNAs and 144 unicox-miRNAs to obtain 43 common miRNAs (**Figure 4B**).

**Figure 5:**
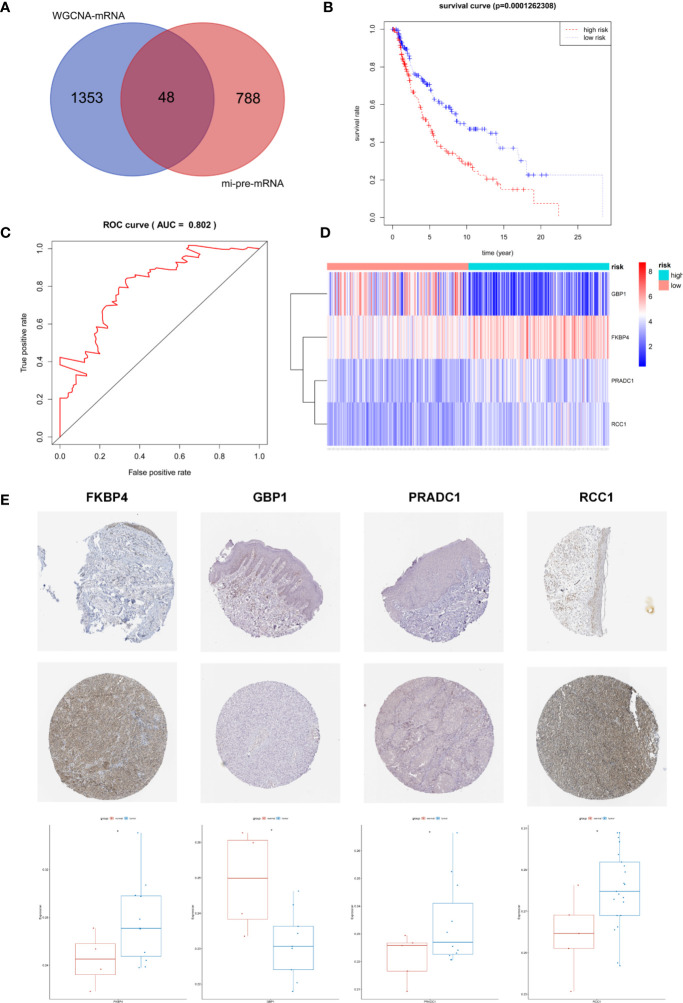
Screening and Identification of Hub Prognostic mRNAs. **(A)** The intersection of mi-pre-mRNAs and WGCNA-mRNAs yields 48 hub mRNAs. **(B)** Survival curve of patients in different risk groups. According to cox regression analysis, the four hub prognostic mRNAs were identified, and the patients were divided into high and low risk groups with median risk value as the boundary. Kaplan-Meier survival analysis showed that the high-risk group had low survival rates (P < 0.001); **(C)** ROC curve was drawn to verify the accuracy of cox survival model, the area under the curve for predicting 5-year survival rate was 0.802, which was with high predictive efficacy. **(D)** Expression heatmap of FKBP4, RCC1, GBP1, and PRADC1 hub prognostic mRNAs between high and low risk groups. **(E)** Immunohistochemical images of the four hub prognostic genes in normal skin and melanoma samples from the Human Protein Atlas database, and gray scale analysis results showed that the expressions of FKBP4, RCC1, and PRADC1 were increased in melanoma and expressions of GBP1 was decreased.

The Targetscan and miRDB databases were employed to predict the mRNAs targeted by these 43 common miRNAs. A total of 836 mi-pre-mRNAs were obtained (**Figures 4C, D**). To screen for mRNAs both involved in the ceRNA network and related to prognosis, we further intersected mi-pre-mRNAs with WGCNA-mRNAs to obtain 48 hub mRNAs (**Figure 5A**).

### Identifying a 4 Hub mRNAs Prognostic Signature by Cox Regression Model

Univariate Cox proportional hazard regression analysis was employed to verify the correlations between expression levels of these 48 common mRNAs and OS of patients, and 13 putative prognostic mRNAs were identified according to the statistical criteria of P < 0.05. These 13 mRNAs were further analyzed by multivariate COX proportional hazard regression analysis, and finally, four hub mRNAs that could be used to predict melanoma prognosis were selected: PRADC1, FKBP4, RCC1, and GBP1 (**Table 4**). To verify the accuracy of this model, Kaplan-Meier survival analysis of high and low risk groups was conducted and the result showed that the survival rate of the high-risk group was significantly lower than that of the low-risk group (P < 0.001, **Figure 5B**). We constructed ROC curves based on the risk scores obtained from the multivariate Cox regression. The area under the 5-year survival curve (AUC) was 0.802, indicating high accuracy and specificity (**Figure 5C**). The heatmap of the four hub mRNAs between the high and low risk groups is shown in **Figure 5D**.

**Table 4:** Identification of hub prognostic mRNAs through univariate cox regression-lasso regression-multivariate cox regression.

Gene	Univariate cox		Multivariate cox
	HR	P value	P value
HLA-DPA1	0.834777173	4.91853E-05	
GBP1	0.828322452	5.96515E-05	0.00286
FGL2	0.818257808	0.000263312	
LCP2	0.76390635	0.000386516	
FKBP4	1.476181993	0.001150697	0.11045
RCC1	1.449636293	0.002269604	0.07937
IFIT3	0.832629786	0.00430891	
C1R	0.860621712	0.009072158	
PRADC1	1.356810513	0.009434333	0.08149
ATP1B1	0.882858597	0.016349185	
PJA2	0.785871702	0.02555629	
RDX	0.817469181	0.03649207	
SLC25A3	1.419662627	0.047260116	

We obtained immunohistochemical images of four hub mRNAs translated proteins in normal skin tissues and melanoma tissues from the HPA database. The gray scale quantification of these images showed that the expression of FKBP4 and RCC1 was significantly upregulated in melanoma, while the expression of PRADC1 was slightly upregulated, and the expression of GBP1 was downregulated (P < 0.05, **Figure 5E**).

**Figure 6:**
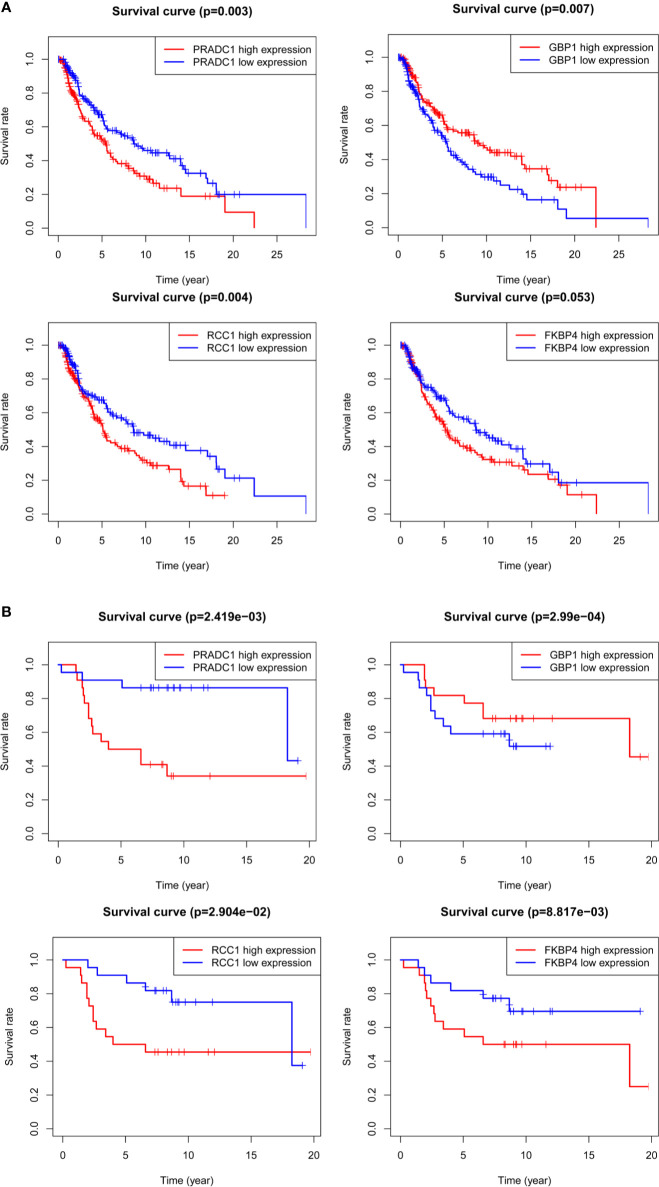
Kaplan-Meier survival analysis for the 4 hub mRNAs with TCGA and GSE98394 mRNAs data. **(A)** Survival curves of four hub mRNAs with TCGA dataset: Patients with high expression of PRADC1 (P = 0.003), RCC1 (P = 0.004), and FKBP4 (P = 0.053) had low survival rate, while patients with low expression of GBP1 had low survival rate (P = 0.007). **(B)** Survival curves of four hub mRNAs with GSE98394 dataset: Patients with high expression of PRADC1 (P = 0.002), RCC1 (P = 0.029), and FKBP4 (P = 0.0008) had low survival rates, and patients with low expression of GBP1 had low survival rates (P = 0.0002).

Kaplan-Meier survival analysis was conducted for the four hub mRNAs and survival curves were drawn with TCGA and GSE98394 mRNAs data. The results of TCGA data (**Figure 6A**) showed that patients with high expression of PRADC1 (P = 0.003), RCC1 (P = 0.004), and FKBP4 (P = 0.053) had low survival rates, and patients with low expression of GBP1 had low survival rates (P = 0.007). Meanwhile, the verification of survival analysis in GSE98394 dataset was completely consisting with the TCGA results (**Figure 6B**). Patients with high expression of PRADC1 (P = 0.002), RCC1 (P = 0.029), and FKBP4 (P = 0.0008) had low survival rates, and patients with low expression of GBP1 had low survival rates (P = 0.0002).

### The Four Core Prognostic mRNAs Are Highly Correlated With Prognostic Clinical Information

**Figure 7:**
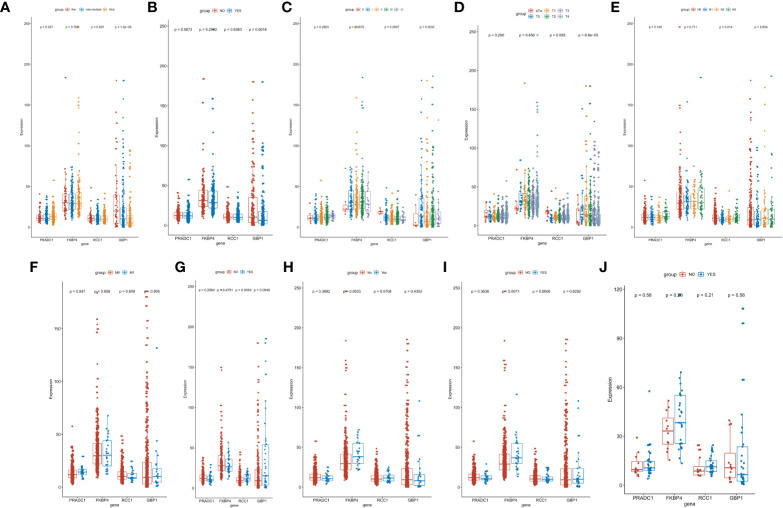
Identification of the difference of hub prognostic mRNAs’ expression between different clinic phenotype and treatment subgroups. **(A)** The expression of GBP1 (P < 0.0001) and PRADC1 (P = 0.037) differed among different Breslow depth. **(B)** The expression of GBP1 was downregulated in melanoma samples with ulcers (P = 0.0016). **(C, D)** The expression of GBP1 showed a downward trend with the increase of total melanoma stage and T stage (both P value <0.01). **(E)** The expression level of RCC1 showed an upward trend with the increase of N stage (P = 0.014). **(F)** The expression of PRADC1 was upregulated in M1 stage samples (P = 0.047). **(G)** The expression of RCC1 was higher in samples after radiotherapy (P = 0.0054). **(H, I)** The expression of FKBP4 was higher in samples previously received neoadjuvant therapy (P = 0.0033) and systemic therapy (P = 0.0071). **(J)** There is no significant difference in the expression of four mRNAs between the interferon treatment and non-treatment groups.

We also analyzed the differences of the 4 hub prognostic mRNAs expression levels between different subtypes with different TNM stages, total melanoma stages, Breslow thickness, ulcerations, neoadjuvant chemotherapy history, interferon treatment history, systemic system treatment history, and radiotherapy history after diagnosis. The results confirmed that the 4 hub prognostic mRNAs differed among subgroups of different disease states and treatment methods. The expression of GBP1 decreased with the increase of tumor depth (P < 0.0001), and the expression of PRADC1 was the highest in thick melanoma subgroup (P = 0.037, **Figure 7A**). The expression of GBP1 in melanoma samples with ulcers was lower than that in the non-ulcer group (P = 0.0016, **Figure 7B**). The expression of GBP1 showed a downward trend with the increase of total melanoma stage and T stage (P < 0.01, **Figures 7C, D**). The expression level of RCC1 showed an upward trend with the increase of N stage (P = 0.014, **Figure 7E**). The expression level of PRADC1 in samples with M1 stage was higher than samples with M0 stage (P = 0.047, **Figure 7F**). In the subgroups with different treatment, the expression of RCC1 in samples after radiotherapy was higher than patients who did not received radiotherapy after diagnosis (P = 0.0054, **Figure 7G**). The expression of FKBP4 was higher in samples previously received neoadjuvant therapy (P = 0.0033, **Figure 7H**) and systemic therapy (P = 0.0071, **Figure 7I**). There is no significant difference in the expression of 4 mRNAs between the interferon treatment and non-treatment groups (**Figure 7J**). These analysis results were consisting with the Cox regression and survival analysis, indicating that the prognostic mRNAs were highly correlation with melanoma outcome and even have the guidance value to treatment choice in some extent.

### Construction of Melanoma Prognostic lncRNA-miRNA-mRNA ceRNA Regulatory Network

**Figure 8:**
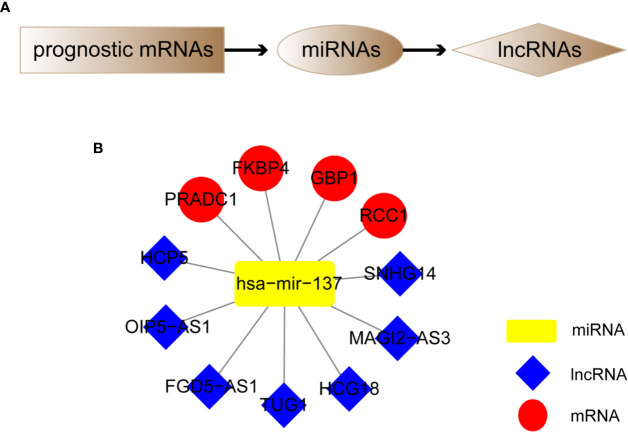
Construction of melanoma prognostic ceRNA network. **(A)** Flowchart of constructing ceRNA network based on four hub prognostic mRNAs. **(B)** Melanoma prognostic ceRNA regulatory networks for seven lncRNAs, one miRNA, and four mRNAs were constructed.

We screened out 4 hub prognostic mRNAs: PRADC1, FKBP4, RCC1, and GBP1. According to the predicted miRNA-mRNA targeting relationship, we identified 1 miRNA: hsa-mir-137. According to the predicted lncRNA-miRNA targeting relationship, 7 lncRNAs that can bind to hsa-mir-137 were matched: HCP5, SNHG14, FGD5-AS1, HCG18, MAGI2-AS3, OIP5-AS1, and TUG1 (**Figure 8A**). The melanoma prognostic ceRNA regulatory network composed of 7 lncRNAs, 1 miRNA, and 5 mRNAs was finally constructed (**Figure 8B**).

### Prognostic ceRNA Network Was Closely Correlated With TIME Verified by Comprehensive Analysis

**Figure 9:**
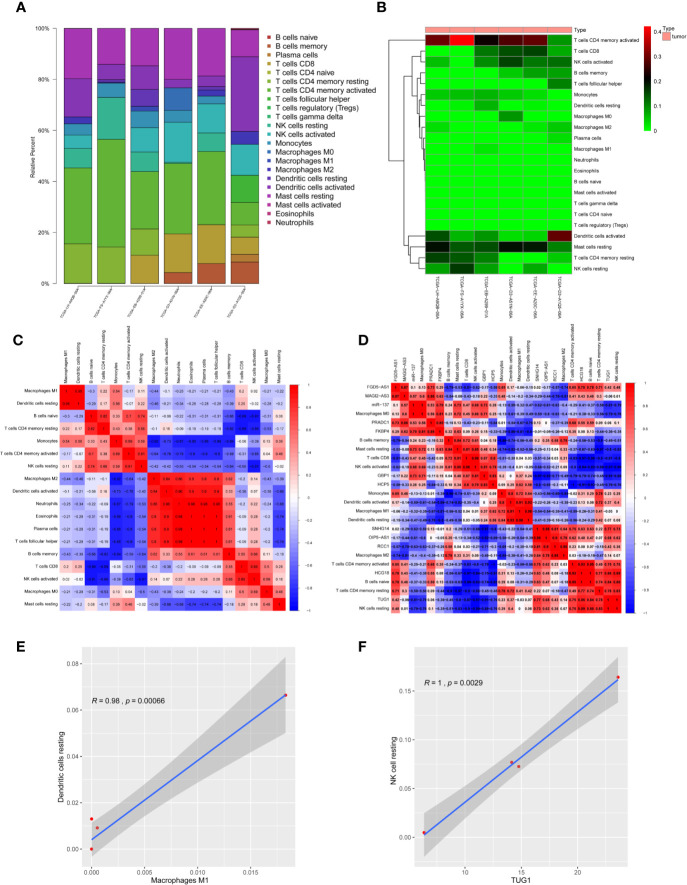
Correlation between melanoma prognosis ceRNA network and TIME. **(A)** Histogram of infiltration ratio of 22 immune cells in melanoma microenvironment. **(B)** Heat map of infiltration ratio of 22 immune cells in melanoma microenvironment. **(C)** Correlation heatmap of infiltrating immune cells. There were significant positive correlations among M2-type macrophages, activated dendritic cells, neutrophils, eosinophilic cells, plasma cells and helper T cells, and M1-type macrophages and resting dendritic cells. **(D)** Correlation heatmap of ceRNAs and infiltrating immune cells. **(E)** Pearson analysis verified that M1-type macrophages were positively correlated with resting dendritic cells in TIME. **(F)** Pearson analysis verified that lnc-TUG1 was positively correlated with NK cells resting in TIME.

CIBERSORT algorithm was used to analyze the infiltrating immune cell components in melanoma samples. In order to fully ensure the accuracy of the results, the analysis was repeated 100 times and only samples with P < 0.05 were adopted for further analysis. The proportion of 22 immune cells in the TIME is shown in **Figures 9A, B**. Excluding four non-infiltrating cells, Pearson correlation analysis was employed to identify the correlation among the remaining 18 microenvironmental immune cells (**Figure 9C**). The results proved the M2 macrophages, activated dendritic cells, neutrophils, eosinophilic cells, plasma cells and helper follicular T cells are positively correlated with each other, and M1 macrophages also have a significant positive correlation with resting dendritic cells (**Figure 9E** and **Supplementary Figures 2** and **3**). At the same time, the correlation between 12 RNAs in ceRNA network and immune cell population was analyzed, and it was found that lncRNA-TUG1 had a significant positive correlation with resting NK cells (**Figures 9D, F**). All the above results proved that the ceRNA network constructed in this study had a certain correlation with the TIME.

**Figure 10:**
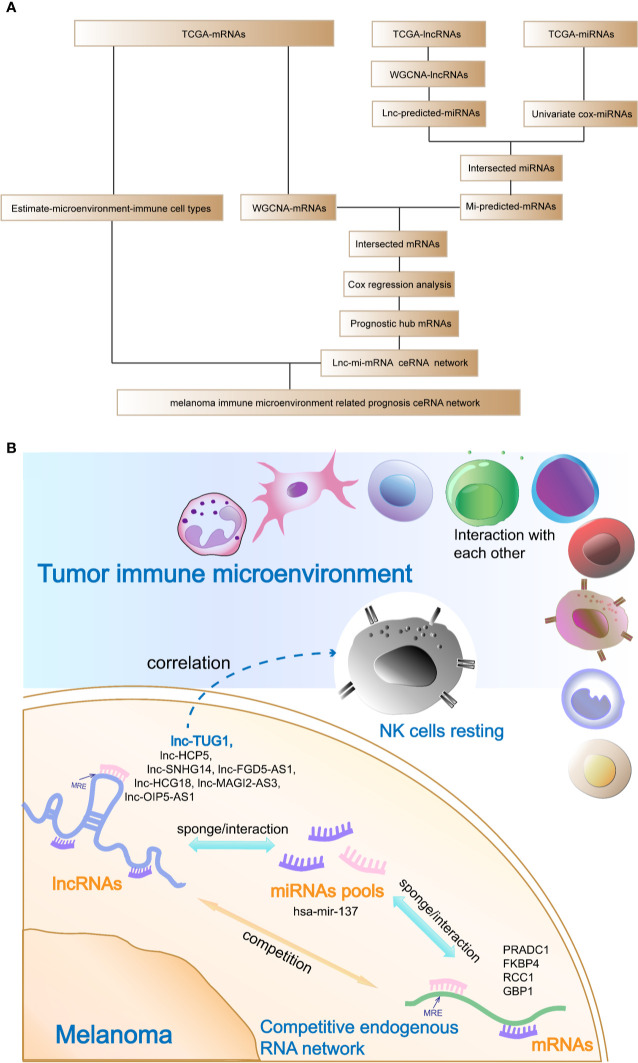
Analysis flow chart and the schematic diagram of this study. **(A)** Analysis flow chart of data obtaining, processing, and verification. **(B)** Schematic diagram of this study: The hub prognostic mRNAs (PRADC1, FKBP4, RCC1, and GBP1) compete to bind hub miRNA- hsa-mir-137 with hub lncRNAs (HCP5, SNHG14, FGD5-AS1, HCG18, MAGI2-AS3, OIP5-AS1, and TUG1), which formed a ceRNA network to regulate the prognosis of melanoma. At the same time, resting NK cells in the tumor immune microenvironment were closely related to the hub prognostic lncRNA-TUG1, indicating that the post-transcriptional regulation might have the potential to remodel tumor microenvironment.

The analysis flow and the schematic diagram of this study is shown in **Figure 10**.

## Discussion

Melanoma is a type of skin malignant tumor which is extremely threatening to human life. Melanoma progresses rapidly and the mortality rate remains high. Although certain success has been reached in immunotherapy and targeted therapy, early removal of local lesions is the effective treatment at present. However, if it is not detected early and surgically removed, melanoma is highly likely to metastasize and has a strong tendency to spread to other parts of the body and causes serious illness and death (31), putting forward higher requirements for early and accurate diagnosis of melanoma based on effective biomarkers (32). At present, the mechanism of melanoma’s pathogenesis, metastasis and recurrence is still unclear, and many researches are devoted to finding new markers as diagnostic and therapeutic targets. The fate of the tumor is determined by both the tumor cells themselves and the microenvironment (33). As a key post-transcriptional regulatory mechanism, ceRNA network has gradually aroused widespread concern for their important roles in melanoma proliferation, apoptosis, and metastasis. Abnormal TIME also promotes immune escape, drug resistance and metastasis of melanoma (34). In this study, we constructed the melanoma prognostic lncRNA-miRNA-mRNA ceRNA network through WGCNA for the first time, and verified the correlation between ceRNA and TIME, excavated The melanoma prognosis-related markers systematically from multi-dimensions of intracell and extra-cell factors and illustrated the pathophysiology of melanoma.

Previous studies have used WGCNA to identify melanoma prognostic mRNAs. Yang et al. obtained the mRNAs expression profiles of melanoma and normal melanocytic nevi from the GEO database, identified the survival-related mRNAs co-expression modules through WGCNA, and screened out 3 hub prognostic mRNAs through survival analysis (STK26, KCNT2, and CASP12) (3). There are also studies constructing melanoma ceRNA networks through identifying the differentially expressed RNAs in melanoma, and analyzing the relationship between immune cell infiltration and melanoma metastasis (15, 35). In any case, our work is the first research to combine WGCNA, Cox regression, ceRNA network, and TIME. First, we use systematically classify gene expression modules and initially screen for survival-related RNAs (**Figures 1** to **3**). Then, we took the intersection of the pre-RNAs and WGCNA-RNAs to further screen hub RNAs to improve the prediction effectiveness (**Figure 4**). Finally, Cox proportional hazards regression model is employed to further identify 4 hub mRNAs and establish the hub prognostic ceRNA network (**Figures 5** and **6**). The above multiple screening processes ensured the accuracy of the hub ceRNA network as a prognostic marker of melanoma. Combining the analysis results of immune cell components in melanoma microenvironment, the prognostic ceRNA network related to melanoma immune microenvironment was outlined (**Figures 7** and **8**).

The abnormal components and functions of immune cells in TIME mediate the immune escape, drug resistance, and metastasis of tumor cells. Tumor cells and the microenvironment respond to each other, promoting the reorganization of innate and acquired immune cells components in the tumor microenvironment, and forming a protective immune network for the tumor: differentiation of macrophages in TIME is abnormal, the anti-tumor M1-macrophages is reduced and inactivated, and the tumor-promoting M2 type is increased (34). In the TIME, NK cell activated receptor expression decreases, immune function of NK cells declines (36). Dendritic cell formation is reduced and tended to be quiescent (37). Low expression of T cell receptor (TCR) and interleukin-2 receptor (IL2-R) in CD8+ T cells inhibits their immune function, and number of tumor-protective regulatory T cells increases (38). All above plays key roles in the process of tumor progression. This study found that there was a positive correlation between lnc-TUG1 in melanoma and NK cells resting, suggesting that lnc-TUG1 highly expressed in melanoma may mediate the inactivation of immune surveillance and immune clearance.

The four hub mRNAs (FKBP4, RCC1, GBP1, and PRADC1), one miRNA (hsa-mir-137), and seven lncRNAs (HCP5, SNHG14, FGD5-AS1, HCG18, MAGI2-AS3, OIP5-AS1, and TUG1) in ceRNA network play important roles in the development of melanoma and other various tumors and are widely involved in the immune regulation process.

The **FKBP4** gene encodes FKBP51 and FKBP52 proteins that can combine with the immunosuppressive protein FK506, which has been confirmed to be involved in the malignant transformation of melanocytes. FKBP51 protein is highly expressed in melanoma and positively correlated with the vertical growth thickness of tumors, which is an independent prognostic indicator of melanoma. Reducing the expression of FKBP51 in melanoma cells can inhibit the cloning formation of tumor cells and reduce the resistance of tumor cells to ionizing radiation (39). What’s more, FKBP4 is widely involved in a variety of immune processes. FKBP51 protein can mediate the inhibition of FK506 on antigen presentation process, and at the same time, its expression is increased in a variety of immune related diseases such as rheumatoid arthritis (40, 41). These reports all support our discovery that the high expression of FKBP4 predicts poor melanoma prognosis, and that FKBP4 is a hub member of TIME-related ceRNA network. **RCC1**, as a guanine nucleotide exchange factor of Ran GTPase, plays a key role in cell mitosis, nucleocytoplasmic transport, and nuclear membrane assembly. Qiao et al. found that in cervical cancer associated with papillomavirus E7, RCC1 expression was significantly increased, and destroyed the normal G1 cell cycle checkpoint by regulating transcription factor E2F1 to accelerate tumor cell proliferation (42). Cekan et al. also confirmed that RCC1 could accelerate the cell cycle and help cells resist senescence caused by DNA damage (43). Therefore, we believe that the role of RCC1 in melanoma may be related to its important positive regulation function of cell cycle, and its increased expression will promote the disordered proliferation of tumor cells, and even mediate the resistance of tumor cells to treatment. **GBP1** encodes ornithine-binding protein, and plays an important role in both immunity and tumor. Studies have shown that melanoma patients with high expression of GBP family, including GBP1, have a good prognosis in the 30-year follow-uperiod, which is consistent with the result in this study that the decline in GBP1 expression predicts poor prognosis of patients (44). Qiu et al. found that inhibiting the expression of GBP1 in macrophages could promote the polarization of macrophages towards a pro-inflammatory phenotype (45). Increased GBP1 expression can inhibit cell proliferation in an inflammatory environment, while down-regulated GBP1 in various tumors such as melanoma, colon cancer, and breast cancer can help protect cells from the damage of inflammatory response (46). All the above studies have confirmed that the hub mRNAs in the prognostic ceRNA network we screened may determine the fate of tumors by regulating melanoma immune microenvironment. However, there is still a lack of research on PRADC1 in tumors or immunity.

In recent years, our understanding of the function of ncRNA has gradually deepened, and a large number of studies have confirmed that miRNA, lncRNA, and other ncRNA play an important role in regulating tumor and immunity. **Hsa-mir-137 (miR-137**) is a recognized tumor suppressor gene, and a large number of studies have confirmed that the low expression of miR-137 is significantly correlated with the poor prognosis of melanoma. miR-137 can bind to various mRNAs as ceRNA, reduce the translation level of mRNA, and regulate the biological process in which it participates. Mir-137 can inhibit the migration and invasion of melanoma cells 45–47 by targeting various mRNAs such as PIK3R3, TBX3, c-Met, YB1, EZH2, and MITF (47–49), and inhibit the proliferation and promote the apoptosis of melanoma cells by competitive binding to genes such as SLC1A5, GLO1, CDK6, etc. (50–52). At the same time, Lv et al. found that mouse microglia could activate IL-10R1 and inhibit the pro-inflammatory factors TNF-α and IL-6 by up-regulating the expression of miR-137 to reduce the inflammatory response (53). These results suggest that miR-137 also has significant immunoregulatory functions.

As a recognized tumor suppressor gene, **lnc-HCP5** can be used as ceRNA to sponge adsorb miR-12 and further promote the expression of RARRES3 gene, thereby inhibiting the growth of melanoma cells. Wei et al. found that the expression of lnc-HCP5 in melanoma decreased and predicted poor prognosis (54). Major histocompatibility complex (MHC) is an important component of the immune response, mainly consisting of MICA and MICB subtypes. The source gene of lnc-HCP5 is located between MICA and MICB genes, which regulates the activities of various immune cells such as B cells, lymphocytes and NK cells, and plays an important role in HIV, AIDS, and various autoimmune diseases (55). Zhao et al. confirmed the role of **lnc-SNHG14** in TIME. They found that in diffuse large B lymphoma, increased expression of lnc-SNHG14 could promote ZEB1 expression through sponge adsorption of miR-5590-3p and further activate PD-L1 and inhibit CD8+ T cells to promote the immune escape of tumor cells (56). Chen et al. found that increasing the expression of **lnc-FGD5-AS1** could reduce the inflammation of periodontal ligament cells caused by lipopolysaccharide through miR-142-3p/SOCS6/NF-κB ceRNA axis reduction (57). As an immune-related gene, **lnc-HCG18** plays an antitumor role in undifferentiated glioma (58). Luan et al. found that the high expression of **lnc-OIP5-AS1** in melanoma could be used as an independent prognostic factor to predict the lower survival rate of melanoma patients, and could sponge adsorb miR-217 as ceRNA to promote glutamine decomposition and accelerate the proliferation rate of melanoma cells (59). Increased expression of **lnc-TUG1** in melanoma promoted tumor growth and metastasis by regulating the TUG1/miR-129-5p/AEG-1 and TUG1/miR-29c-3p/RGS1 ceRNA regulatory axis (60, 61). At the same time, reducing the expression of lnc-TUG1 can also suppress the production and release of various immune factors through the TUG1/miR-9-5p/NF-κB1/p50 axis, thus reducing the the inflammatory response of multiple sclerosis. Our study also found that lnc-TUG1 was significantly correlated with quiescent NK cells, suggesting that lnc-TUG1 had the potential to become a tumor immunotherapy target. To date, there have been no reports on functions of SNHG14, HCG18, and MAGI2-AS3 in melanoma, but they constitute a precisely regulated ceRNA network in various solid tumors such as colon cancer, bladder cancer, liver cancer, etc., and regulate the malignant behavior of tumor cells (62, 63). The above studies all support that the ceRNA network we constructed regulates the function of immune cells in the microenvironment and the immune response of tumors, and may play a key role in the formation and evolution of TIME. Meanwhile, the TIME-related ceRNA network in this study has also been proved to be involved in the regulation of the occurrence and development of melanoma and has high prognostic value.

In any case, the limitations of our study suggest that there is still a long way to go in using bioinformatics methods to comprehensively reveal the biological processes of tumors. First of all, although the role of most RNAs in ceRNA network in melanoma or other tumors has been verified by multiple studies, we still found that miR-137 and some lncRNAs showed no significant statistical difference in survival analysis, which means that the amount of data obtained from the public database is still limited, and may limit the accuracy of analysis to some extent. Although this study used WGCNA, Cox regression, Kaplan-Meier survival analysis, and multiple screening methods that combine multiple intersections to improve the prediction accuracy of TIME-related ceRNA for melanoma prognosis, it was still limited to a certain extent by the relatively insufficient number of public data sets under the current situation. Secondly, this study only verified the correlation between the proportion of TIME immune cells and ceRNA, and did not discuss the effect of TIME on melanoma prognosis in detail. In the future, large amounts of data from multiple platforms will be needed to further explore the relationship between immune infiltration and heterogeneity of TIME and melanoma. Finally, although the role of most ncRNAs in this study have been reported by several research respectively, the regulatory and competitive relationship between the lncRNA-miRNA-mRNA axis in melanoma is still lack of experimental verification. Besides, we only screened out the relationship between ceRNA and melanoma TIME, but there is still a lack of relevant research on how this RNA regulates immune cells in the microenvironment.

## Conclusion

Our research focused on the identification of prognostic genes by WGCNA, combined with cox regression, Kaplan-Meier survival analysis and CIBERSORT cell composition analysis, etc., established a prognostic ceRNA network related to the melanoma TIME for the first time. We revealed the importance of 4 mRNAs (FKBP4, RCC1, GBP 1, and PRADC1), 1 miRNA (hsa-mir-137), and 7 lncRNAs (HCP5, SNHG14, FGD5-AS1, HCG18, MAGI2-AS3, OIP5-AS1, and TUG1) in reshaping the immune microenvironment and regulating the melanoma process, providing new biomarkers for understanding the mechanism of melanoma occurrence and development at multiple levels, and promoting exploration of prognostic and therapeutic targets.

## Data Availability Statement

All datasets presented in this study are included in the article/**Supplementary Material**.

## Author Contributions

Conceptualization: YC and CXL. Methodology: CXL, SW, and LJ. Software: XW, QW, and XS. Validation: XW, YRL, and LY. Formal analysis: YC and YS. Investigation: YL, CYL, and CL. Resources: XW and ZW. Writing (original draft preparation): YC and XW. Writing (review and editing): XW and ZW. All authors contributed to the article and approved the submitted version.

## Funding

This work was supported by The National Key R&D program of China (2018YFC1106000).

## Conflict of Interest

The authors declare that the research was conducted in the absence of any commercial or financial relationships that could be construed as a potential conflict of interest.
