# Supplementary material for: Immune Microenvironment Related Competitive Endogenous RNA Network as Powerful Predictors for Melanoma Prognosis Based on WGCNA Analysis
Source: Front Oncol. 2020 Oct 27;10:577072. doi: 10.3389/fonc.2020.577072 (PMC7653056; doi:10.3389/fonc.2020.577072)
Supplement: Supplementary file 2 [file Table_1.docx]

**Supplementary Material:**

**Supplementary Figures:**

**
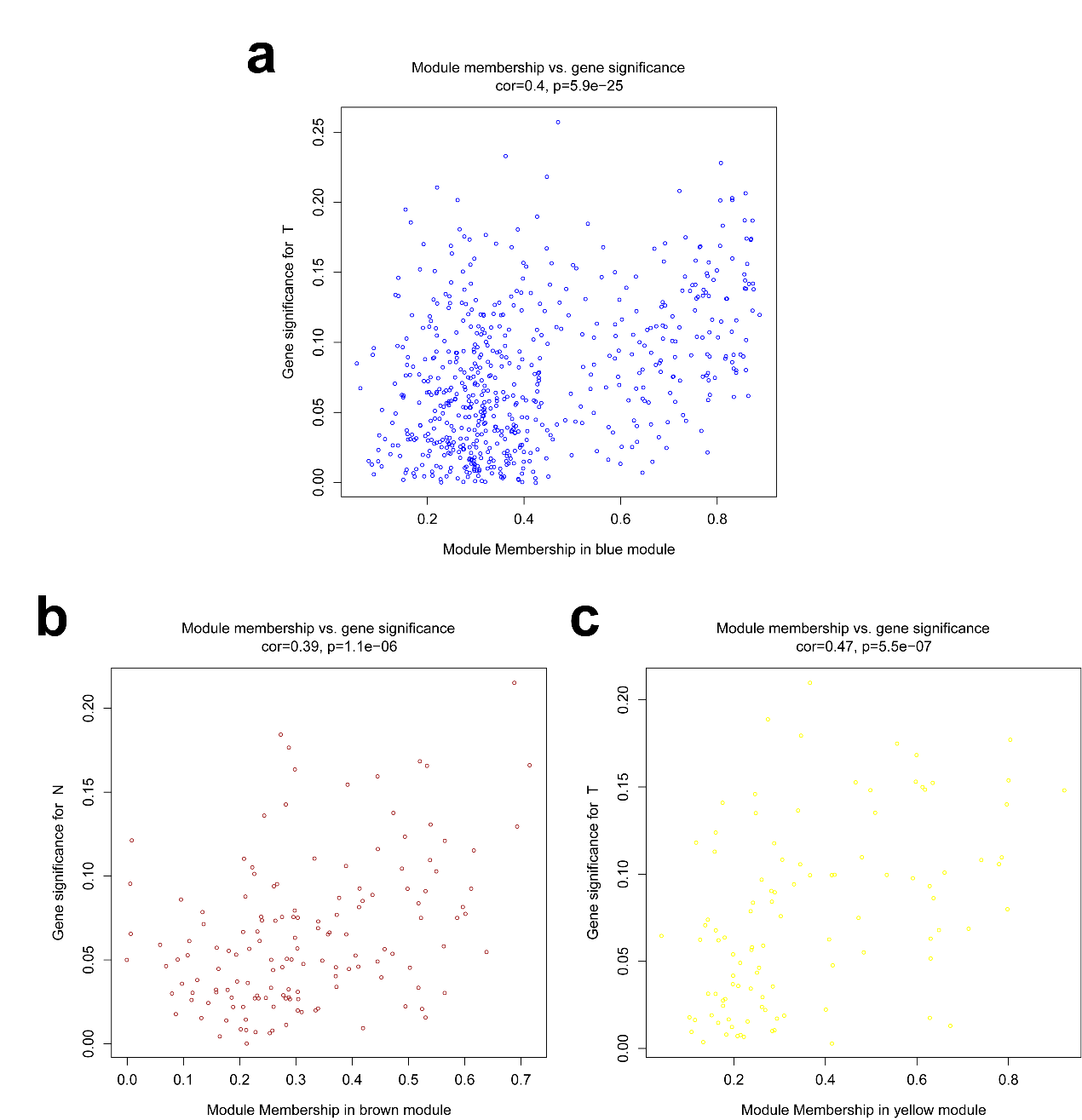
**

**Supplementary Figure 1. The relationship between mRNAs and lncRNAs modules and clinical traits in WGCNA analyzed.** (a) The blue module of mRNAs was significantly correlated with T staging (in situ infiltration) of SCM; (b) The brown module of lncRNAs was significantly correlated with N staging (lymph node infiltration) of SCM; (c) The yellow module of lncRNAs was significantly correlated with T staging (in-situ infiltration) of SCM.

**
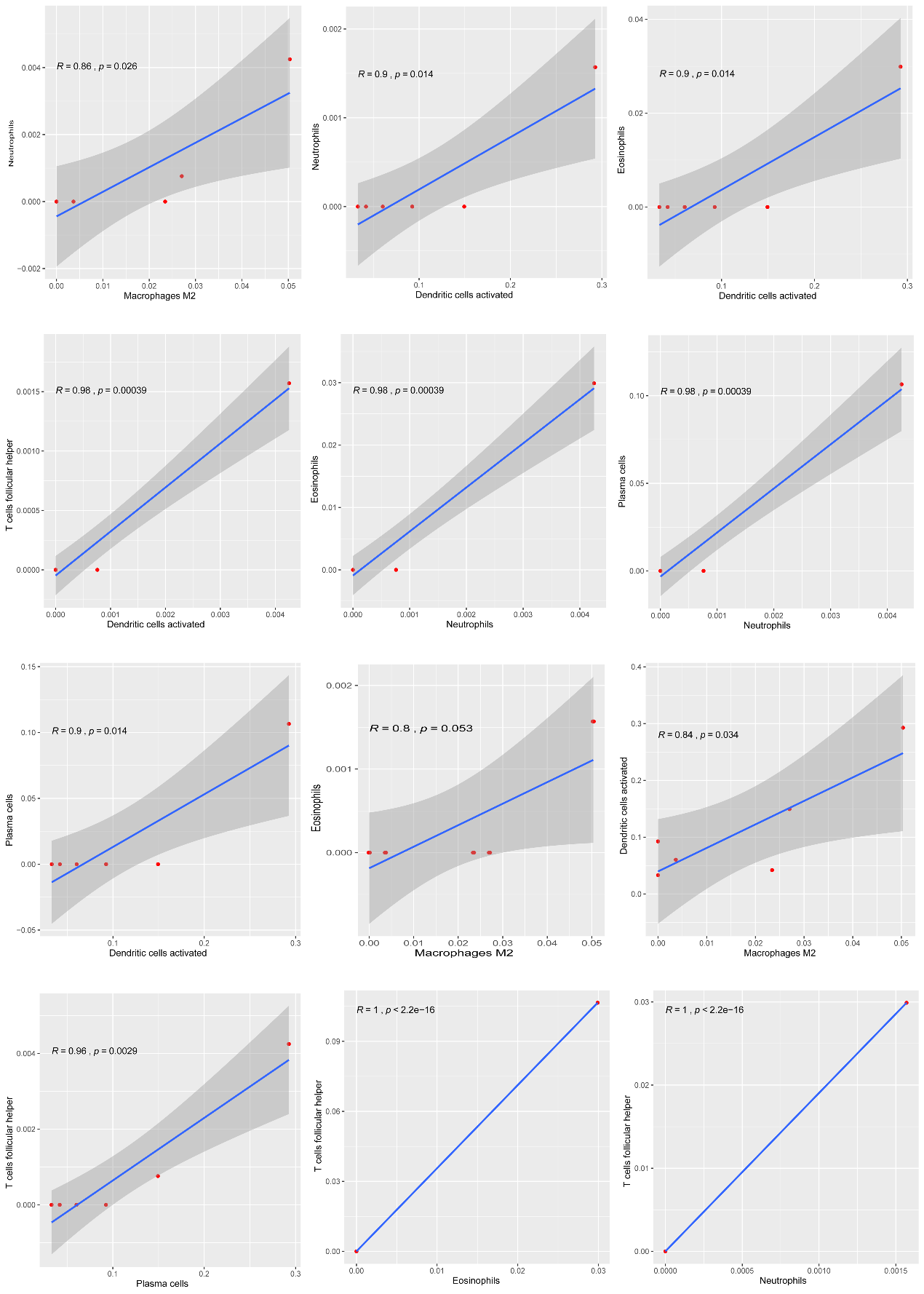
**

**Supplementary Figure 2.** **Verification of correlation between various immune cells in SCM tumor immune microenvironment.**

**
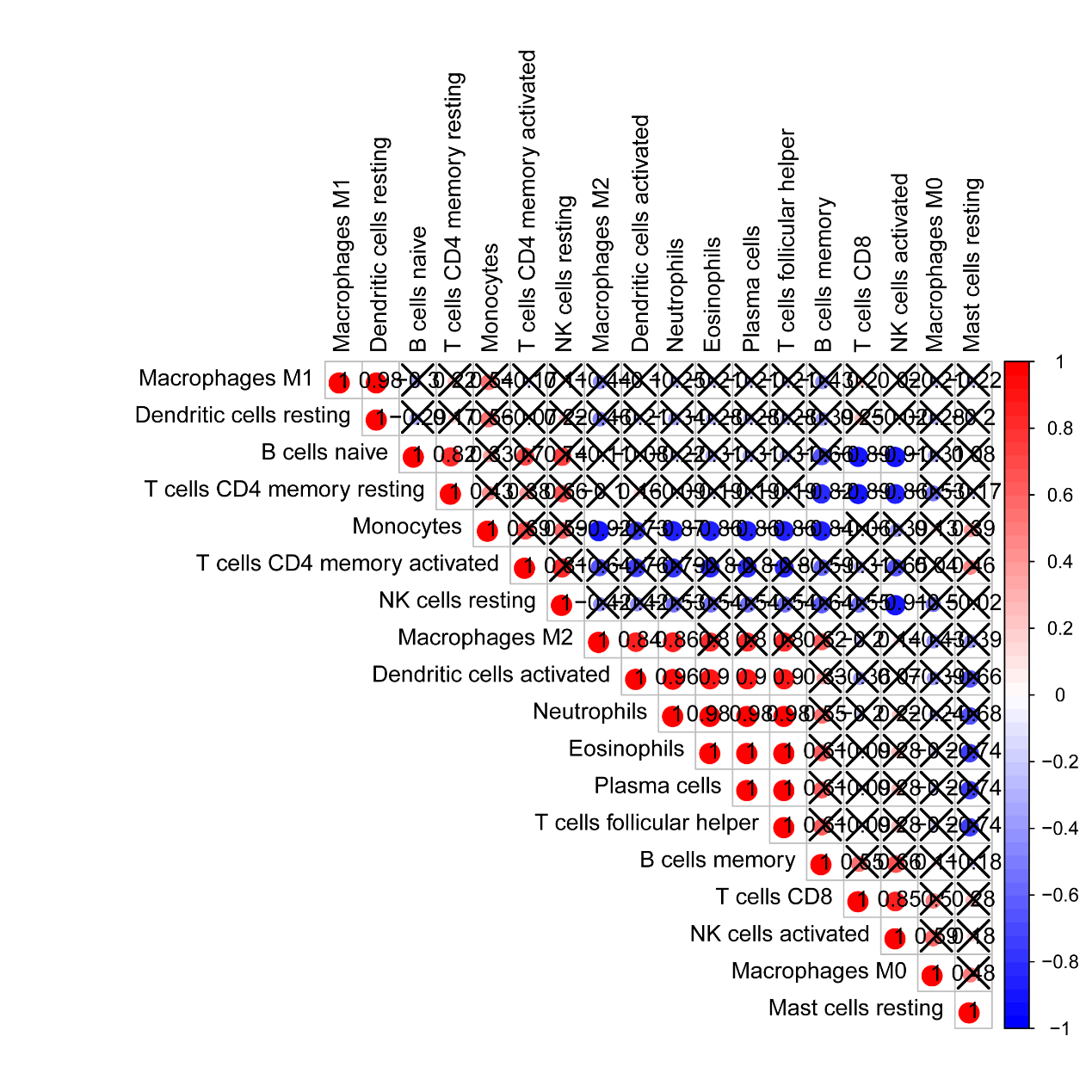
**

**Supplementary Figure 3. Correlation heatmap of various immune cells in the SCM immune microenvironment.** The red circle represents a positive correlation and the blue circle represents a negative correlation. The darker the color, the higher the correlation. Crossed circles represent no correlation, and the numbers on the circles are correlation coefficients.

**Supplementary Table 1.** **144 prognostic miRNAs screened by univariate Cox proportional hazard regression**

| **gene** | **P value** | **gene** | **P value** | **gene** | **P value** | **gene** | **P value** | **gene** | **P value** |
| --- | --- | --- | --- | --- | --- | --- | --- | --- | --- |
| hsa-mir-625 | 2.51E-06 | hsa-mir-3127 | 0.00330724 | hsa-mir-375 | 0.010203391 | hsa-mir-3691 | 0.021820895 | hsa-mir-1224 | 0.036984782 |
| hsa-mir-29c | 9.39E-06 | hsa-mir-4524a | 0.003374323 | hsa-mir-5090 | 0.010757729 | hsa-mir-219a-1 | 0.022193956 | hsa-mir-514a-3 | 0.037232976 |
| hsa-mir-100 | 2.57E-05 | hsa-mir-548ao | 0.003386973 | hsa-mir-29b-1 | 0.010766365 | hsa-mir-20a | 0.022291739 | hsa-mir-151b | 0.037591655 |
| hsa-mir-4461 | 3.13E-05 | hsa-mir-4785 | 0.003465147 | hsa-mir-320a | 0.011160251 | hsa-mir-582 | 0.022564018 | hsa-mir-3144 | 0.037845592 |
| hsa-mir-155 | 8.18E-05 | hsa-mir-6125 | 0.003474202 | hsa-mir-1914 | 0.011266851 | hsa-let-7b | 0.022694707 | hsa-mir-4999 | 0.038392918 |
| hsa-mir-573 | 0.000125756 | hsa-mir-2110 | 0.003738153 | hsa-mir-204 | 0.012592558 | hsa-mir-8072 | 0.023200068 | hsa-mir-5002 | 0.038933002 |
| hsa-mir-7702 | 0.000140946 | hsa-mir-3202-1 | 0.003887556 | hsa-mir-505 | 0.01270623 | hsa-mir-320c-2 | 0.023224203 | hsa-mir-301b | 0.039467004 |
| hsa-mir-1976 | 0.000197107 | hsa-mir-548b | 0.004060996 | hsa-mir-4444-2 | 0.01292597 | hsa-mir-6884 | 0.024219042 | hsa-mir-202 | 0.039684665 |
| hsa-mir-3667 | 0.000219973 | hsa-mir-29b-2 | 0.004444493 | hsa-mir-135a-2 | 0.013309443 | hsa-mir-7706 | 0.025412222 | hsa-mir-514a-1 | 0.039748975 |
| hsa-mir-6842 | 0.000270192 | hsa-mir-18a | 0.004516361 | hsa-mir-504 | 0.013637383 | hsa-mir-592 | 0.025982213 | hsa-mir-4453 | 0.039859605 |
| hsa-mir-342 | 0.000433902 | hsa-mir-4491 | 0.004591642 | hsa-mir-146b | 0.013796471 | hsa-mir-584 | 0.026073563 | hsa-mir-137 | 0.039942902 |
| hsa-mir-150 | 0.000619914 | hsa-mir-205 | 0.004673892 | hsa-mir-3690-1 | 0.014700745 | hsa-mir-129-2 | 0.026298852 | hsa-mir-514a-2 | 0.042342736 |
| hsa-let-7g | 0.000689731 | hsa-mir-26a-1 | 0.004709205 | hsa-mir-3680-2 | 0.015661414 | hsa-mir-4519 | 0.026876811 | hsa-mir-466 | 0.042835776 |
| hsa-mir-6715a | 0.00072498 | hsa-mir-26a-2 | 0.004760644 | hsa-mir-4691 | 0.015996445 | hsa-mir-507 | 0.027282194 | hsa-mir-1276 | 0.044017329 |
| hsa-mir-3170 | 0.000752197 | hsa-mir-6891 | 0.004896859 | hsa-mir-19a | 0.016204827 | hsa-mir-892a | 0.027307455 | hsa-mir-6803 | 0.045757572 |
| hsa-mir-5571 | 0.000849491 | hsa-mir-3200 | 0.005615647 | hsa-mir-5586 | 0.016273006 | hsa-mir-521-1 | 0.027448197 | hsa-mir-5690 | 0.046706644 |
| hsa-mir-5091 | 0.000859357 | hsa-mir-3150b | 0.005846924 | hsa-mir-548k | 0.016807003 | hsa-mir-767 | 0.02863161 | hsa-mir-6720 | 0.047658614 |
| hsa-mir-203b | 0.000873939 | hsa-mir-511 | 0.006693011 | hsa-mir-129-1 | 0.017923109 | hsa-let-7e | 0.029817493 | hsa-mir-6892 | 0.048180494 |
| hsa-mir-125b-1 | 0.001188951 | hsa-mir-140 | 0.006974415 | hsa-mir-181a-2 | 0.018564976 | hsa-mir-497 | 0.03061646 | hsa-mir-651 | 0.048611249 |
| hsa-mir-4434 | 0.001199748 | hsa-mir-1181 | 0.007366326 | hsa-mir-7846 | 0.018693329 | hsa-mir-142 | 0.030978195 | hsa-mir-105-1 | 0.049113759 |
| hsa-mir-1910 | 0.001439802 | hsa-mir-7974 | 0.007471385 | hsa-mir-4772 | 0.018755431 | hsa-mir-5699 | 0.031246948 | hsa-mir-4766 | 0.049227251 |
| hsa-mir-125b-2 | 0.001670081 | hsa-mir-4739 | 0.007916793 | hsa-mir-320c-1 | 0.019221916 | hsa-mir-4800 | 0.03127945 | hsa-mir-3184 | 0.049428881 |
| hsa-let-7a-2 | 0.001890148 | hsa-mir-6851 | 0.008122833 | hsa-mir-3928 | 0.01959855 | hsa-mir-521-2 | 0.031933454 | hsa-mir-4791 | 0.049698135 |
| hsa-let-7a-3 | 0.001922408 | hsa-mir-605 | 0.00823567 | hsa-mir-17 | 0.019625255 | hsa-mir-6503 | 0.033915044 | hsa-mir-6783 | 0.04988614 |
| hsa-let-7a-1 | 0.001924702 | hsa-mir-147b | 0.008336957 | hsa-mir-943 | 0.02003626 | hsa-mir-144 | 0.034810658 |  |  |
| hsa-mir-361 | 0.002095041 | hsa-mir-3680-1 | 0.008532045 | hsa-mir-5193 | 0.020266562 | hsa-mir-4657 | 0.034981856 |  |  |
| hsa-mir-1286 | 0.002308339 | hsa-mir-551a | 0.008533213 | hsa-mir-423 | 0.020919354 | hsa-mir-520f | 0.035160711 |  |  |
| hsa-mir-3615 | 0.002656531 | hsa-mir-3605 | 0.008631741 | hsa-mir-3166 | 0.020974379 | hsa-mir-195 | 0.035328175 |  |  |
| hsa-mir-4522 | 0.002966066 | hsa-mir-642a | 0.009686112 | hsa-mir-4521 | 0.021703057 | hsa-mir-6813 | 0.035463234 |  |  |
| hsa-mir-6510 | 0.003255624 | hsa-mir-211 | 0.010153263 | hsa-mir-4442 | 0.02179961 | hsa-mir-6501 | 0.036146309 |  |  |
